# Synthesis of Macroporous Magnetic Fe_3_O_4_ Microparticles Via a Novel Organic Matter Assisted Open-Cell Hollow Sphere Assembly Method

**DOI:** 10.3390/ma11091508

**Published:** 2018-08-23

**Authors:** Huixia Wang, Ximing Pu, Yaquan Zhou, Xianchun Chen, Xiaoming Liao, Zhongbing Huang, Guangfu Yin

**Affiliations:** College of Materials Science and Engineering, Sichuan University, Chengdu 610065, China; whx_93@163.com (H.W.); puximing@163.com (X.P.); zhouyaquan@163.com (Y.Z.); chenxianchun@scu.edu.cn (X.C.); sherman_xm@163.com (X.L.); zbhuang@scu.edu.cn (Z.H.)

**Keywords:** macroporous Fe_3_O_4_ microparticles, open-cell hollow microsphere, nanoscale Kirkendall effect, 1-vinyl-2-pyrrolidinone, 2-acrylamido-2-methyl propane sulfonic acid

## Abstract

Macroporous magnetic Fe_3_O_4_ microparticles, which might act as both drug carriers and magnetocaloric media, were expected to have broad application prospects on magnetocaloric-responsively controlled drug release systems. A kind of macroporous magnetic Fe_3_O_4_ microparticle was prepared by an organic matter assisted open-cell hollow sphere (hollow sphere with holes on shell) assembly method in this study. 1-vinyl-2-pyrrolidinone (NVP) and 2-acrylamido-2-methyl propane sulfonic acid (AMPS) were selected as the template and the binder, respectively. Ferrous ions were specifically bound to carbonyl groups on NVP and were then reduced by NaBH_4_. The reduced irons underwent heterogeneous nucleation and grain growth to form Fe^0^/Fe_3_O_4_ microspheres consisting of a lot of nano-Fe^0^ grains, and were then assembled into Fe^0^/Fe_3_O_4_ microparticles wrapped by AMPS. Results indicate that NVP binding with ferrous ions can promote a self-polymerization process and the formation of Fe^0^/Fe_3_O_4_ microspheres, while AMPS enwrapping around the resultant microspheres can facilitate their assembly into larger aggregates. As a result, macroporous Fe_3_O_4_ microparticles composed of several open-cell hollow Fe_3_O_4_ microspheres can be obtained under a Kirkendall-controlled oxidation. Moreover, these as-prepared macroporous Fe_3_O_4_ microparticles possess a narrow particle size distribution and exhibit ferromagnetism (Ms = 66.14 emu/g, Mr = 6.33 emu/g, and Hc = 105.32 Oe). Our work, described here, would open up a novel synthesis method to assemble macroporous magnetic Fe_3_O_4_ microparticles for potential application in magnetocaloric-responsively controlled drug release systems.

## 1. Introduction

Nowadays, the nanoscale/microscale porous particles of transition metal oxides have attracted great attention due to their specific optical, electrical, and magnetic performances derived from the d-layer orbitals with unfilled valence, as well as their unique absorptivity, penetrability, and chemical activity—resulting from their porous structure [1,2,3,4]. Among them, the porous magnetite (ferroferric oxide, Fe_3_O_4_, one of the transition metal oxides) has been widely applied in catalysis, electrode, microwave absorption, and separation, owing to its low cytotoxicity, adjustable magnetism, high loading capacity, and long circulation [5,6,7,8].

Depending on the pore size, porous materials can be classified into microporous (pore diameter < 2 nm), mesoporous (2–50 nm), and macroporous materials (>50 nm). Microporous material notes were easy to get and are not the focus of our attention. Mesoporous materials have emerged as the most widely studied porous materials in recent years, and various methods for preparing mesoporous materials have been reported [9,10,11,12,13]. However, there are few studies on macroporous materials, which have larger pore diameters and possess a wider application prospect than mesoporous materials in the field of loading and the transport of large-sized particles, especially organic nanoparticles. For instance, macroporous Fe_3_O_4_ micro/nanoparticles are able to act as carriers for loading and transporting drug-loaded temperature-sensitive micelles in magnetocaloric-responsively controlled drug release systems. On the one hand, macroporous Fe_3_O_4_ micro/nanoparticles would improve the drawbacks of drug loss in micelles, and on the other hand, they would also provide a heat source for the control of temperature-sensitive materials. Hence, the development of macroporous Fe_3_O_4_ micro/nanoparticles is of importance to achieve an effective and controlled release of drugs [14]. At present, the preparations of macroporous materials mostly rely on adopting the colloidal crystal template method. However, the colloidal crystal template is usually obtained by the self-assembly of organic microspheres, and the size of the template is far exceeded by the micron scale, thus leading to the colloidal crystal template method being just as suitable for the preparation of the macroporous bulk materials—rather than the macroporous microparticles [15,16,17]. Therefore, there is, so far, still a lack of an efficient synthesis approach to prepare macroporous transition metal oxides-based microparticles.

In recent years, various hollow nanospheres have been reported in succession [18,19,20], and some nano or submicron scale hollow Fe_3_O_4_ spheres have been prepared through the template method [21], the hydrothermal method [22], the self-assembly method [23], and the controlled oxidation method [24]. Among these hollow spheres synthesis methods, the controlled oxidation method, based on the nanoscale Kirkendall effect (NKE), has attracted widespread attention because of its controllability in outer and inner diameters of hollow spheres. The Kirkendall controlled oxidation method is involved in two main processes. The first process is the synthesis of Fe^0^/Fe_3_O_4_ particles, in which the Fe^0^ particles are first synthesized and then the Fe^0^/Fe_3_O_4_ core-shell particles are formed after the surficial oxidation is finished. The second process is the oxidation and cavitation of Fe^0^/Fe_3_O_4_ particles, in which case the Fe atoms in the core preferentially diffuse to the surface at the elevated temperature under the action of NKE, thus resulting in a hollowing Fe_3_O_4_ structure [25,26,27]. The morphology and size of the Fe^0^/Fe_3_O_4_ core-shell particles, formed in the first step, determine the size and pore diameter of the final hollow Fe_3_O_4_ spheres. As the spheres are gradually hollowed during the oxidation process, some holes might appear on the shell to form the open-cell hollow sphere (hollow sphere with holes on the shell).

Based on the open-cell hollow nanospheres, a potential strategy of assembling the open-cell hollow microspheres/nanospheres into macroporous microparticles (the aggregate of open-cell hollow microspheres/nanospheres in micron/submicron scale) is proposed in our research. There are two paths to assemble hollow spheres into macroporous aggregates, including the hollowing-first method and the assembling-first method. Some troubles might exist in the direct assembly of the open-cell hollow Fe_3_O_4_ microspheres into macroporous aggregates because the holes on the shell is probably sealed up by the binder, and the aggregation of open-cell hollow Fe_3_O_4_ microspheres is difficult to maintain once the binder is removed through calcination. In contrast, it would be practicable that the Fe^0^/Fe_3_O_4_ microspheres are pre-assembled into aggregates and then oxidized and hollowed in consideration of the local fusion of adjacent microspheres, which are the result of the grain growth during the oxidation process that is propitious to the maintenance of macroporous aggregates.

In this study, a kind of macroporous magnetic Fe_3_O_4_ microparticles, composed of Fe_3_O_4_ open-cell hollow microspheres with sizes ranging from several hundred nanometers to several microns, were prepared by a novel organic matter assisted open-cell hollow sphere assembly method, in which 1-vinyl-2-pyrrolidinone (NVP) and 2-acrylamido-2-methyl propane sulfonic acid (AMPS) were selected as the template and the binder, respectively. The effects of NVP and AMPS additions on the composition and morphology of the Fe^0^/Fe_3_O_4_ microspheres and the corresponding aggregates were explored in detail. Moreover, the regulatory mechanisms of NVP and AMPS on the formation and assembly of Fe^0^/Fe_3_O_4_ microspheres were preliminary discussed. In addition, the morphology, pore structure, and magnetic performance of macroporous Fe_3_O_4_ aggregates upon the Kirkendall controlled oxidation were also investigated. The results show that the macroporous Fe_3_O_4_ microparticles, in the micron scale, were prepared by the assembly of the Fe_3_O_4_ open-cell hollow microspheres—in the assembling-first way—whilst the organic monomers of NVP and AMPS played a crucial role in the formation of the Fe^0^/Fe_3_O_4_ microspheres and their assembly into Fe^0^/Fe_3_O_4_ microparticles.

## 2. Materials and Methods

### 2.1. Raw Materials and Reagents

Two kinds of organic monomers, 1-vinyl-2-pyrrolidinone (C_6_H_9_NO, NVP) with 99.5% purity that is stabilized with 4-Methoxyphenol (MEHQ), and 2-acrylamido-2-methyl propane sulfonic acid (C_7_H_13_NO_4_S, AMPS) with 98% purity, were purchased from J & K Scientific Ltd., Beijing, China. Trimethylamine N-oxide (C_3_H_9_NO, TMAO) with 98% purity was the oxygen transfer agent used in the solvothermal reaction and was purchased from Sigma Aldrich Trading Ltd., Burlington, MA, USA. Iron chloride tetrahydrate (FeCl_2_·4H_2_O) and sodium borohydride (NaBH_4_), as well as other conventional reagents were analytical reagents without further purification and were purchased from Kelong Chemicals, Chengdu, China. All reagents were used without further purification.

### 2.2. Synthesis of Fe^0^/Fe_3_O_4_ Microparticles

In a typical synthesis, 0.05 g AMPS, 25 μL NVP, and 1.243 g iron chloride tetrahydrate (FeCl_2_·4H_2_O) was dissolved in 50 mL aqueous under mechanical stirring, followed by 30 min ultrasonic oscillation for complete dissolution. 20 mL 0.54 mol/L NaBH_4_ aqueous solution was slowly dripped into the FeCl_2_ solution under vigorously mechanical agitation. After the completion of the dripping, agitation was continued for 30 min. The precipitate was washed with deionized water and ethanol several times, and then vacuum dried at room temperature for 8 h to obtain the Fe^0^/Fe_3_O_4_ microparticles marked as P^0.05^_25_. The superscript indicated the quality of AMPS in grams and the subscripts indicated the volume of NVP in microliters. A series of experiments based on the control variable method were conducted to investigate the effects of the two organic matters on the formation of the Fe^0^/Fe_3_O_4_ microparticles. In the first group, the AMPS addition was fixed at 0.05 g, and the NVP additions were determined as 0, 12.5, 25, 50, and 100 μL, respectively. While in the second group, the NVP addition was fixed at 25 μL, and the AMPS additions were selected as 0, 0.01, 0.05, 0.5, and 1 g, respectively.

### 2.3. Synthesis of Macroporous Fe_3_O_4_ Microparticles

0.16 g of Fe^0^/Fe_3_O_4_ microparticles P^0.05^_25_ was dispersed in a mixed solution of 55 mL of hexane and 5 mL of ethanol dissolved with 0.06 g of TMAO. Then, the mixed slurry was decanted into a 100-mL Teflon lined stainless steel autoclave, sealed, and preserved at 180 °C for 4 h. After cooling to room temperature, the precipitate was washed by deionized water and ethanol and dried in a vacuum oven for 8 h (marked as I^0.05^_25_). After calcination at 400 °C for 3 h in a nitrogen atmosphere, the macroporous Fe_3_O_4_ microparticles were obtained (marked as F^0.05^_25_).

### 2.4. Characterization

The phase composition of prepared powders was determined by X-ray diffractometer (XRD6100, Shimadzu, Kyoto, Japan) with Cu Kα radiation (λ = 0.154 nm) at a scanning rate of 5°/min for 2θ ranging from 10° to 80° and X-ray photoelectron spectroscope (XPS, Escalab 250Xi, Thermo Fisher Scientific, Waltham, MA, USA). The existence forms of the two organic matters were analyzed by the Fourier transform infrared spectroscopy (FTIR, Nicolet 6700, Bruker Optics, Ettlingen, Germany) using the KBr method. Morphological observation was carried out by scanning electron microscopy (SEM, JSM-7500F, JEOL Ltd., Tokyo, Japan) at a voltage of 15 kV and a low-resolution/high-resolution transmission electron microscopy (TEM/HRTEM, Zeiss Libra 200FE, Carl Zeiss, Jena, Germany) at voltage of 200 kV, respectively. Element distribution was characterized by an energy dispersive X-ray spectroscope (EDXS, Super-X, EDAX Inc., San Diego, CA, USA) attached to a transmission electron microscopy (TEM, Zeiss Libra 200FE, Carl Zeiss, Jena, Germany). The particle size was measured by a laser particle size analyzer (LPSA, Mastersizer 3000E, Malvern Instruments Ltd., Malvern, UK) using water as the dispersant. Nitrogen adsorption-desorption isotherms were obtained with a high performance micropore analyzer (Kubo-X1000, Beijing Biaode Electronic Techology Co. Ltd., Beijing, China) at 77 K. Hysteresis loop was obtained by vibrating sample magnetometer (VSM, Lakeshore 7410, Lake Shore Cryotronics Inc., Columbus, OH, USA) at room temperature.

## 3. Results and Discussion

### 3.1. Composition and Morphology of Fe^0^/Fe_3_O_4_ Microparticles

**Figure 1:**
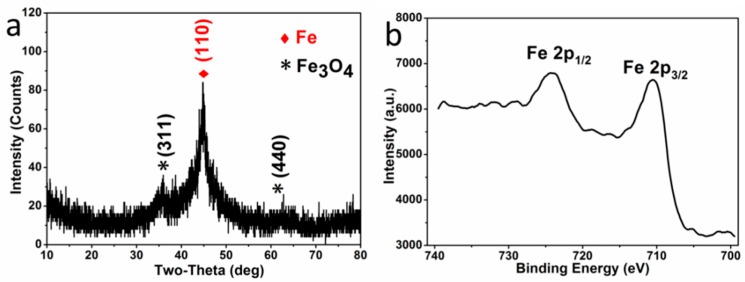
XRD pattern (**a**) and Fe 2p XPS high resolution spectroscopy (**b**) of Fe^0^/Fe_3_O_4_ microparticles P^0.05^_25_.

The crystal phase composition of the microparticles P^0.05^_25_, obtained by reduction, was investigated by XRD and XPS. In the XRD pattern (Figure 1a), the diffraction peaks centered at 44.7° is indexed to the characteristic peaks (110) of Fe^0^ (JCPDS NO. 06-0696), while the peaks centered at 35.5° and 62.6° are indexed to the characteristic peaks (311) and (440) of Fe_3_O_4_ (JCPDS NO. 88-0866), respectively. The characteristic peaks of Fe_2_O_3_ are absent in the XRD pattern, and there are no specific satellite structures of ferric oxide in the high resolution Fe 2p XPS spectroscopy (Figure 1b)—verifying the absence of γ-Fe_2_O_3_ or α-Fe_2_O_3_ [28,29]. Therefore, the oxide in the sample is pure Fe_3_O_4_. Moreover, the characteristic peaks of Fe^0^ are much higher than that of Fe_3_O_4_, indicating that the microparticles are mainly composed of a large amount of Fe^0^ and a small amount of Fe_3_O_4_, resulting from the surficial oxidation in the atmosphere (expressed with Fe^0^/Fe_3_O_4_ microparticles).

**Figure 2:**
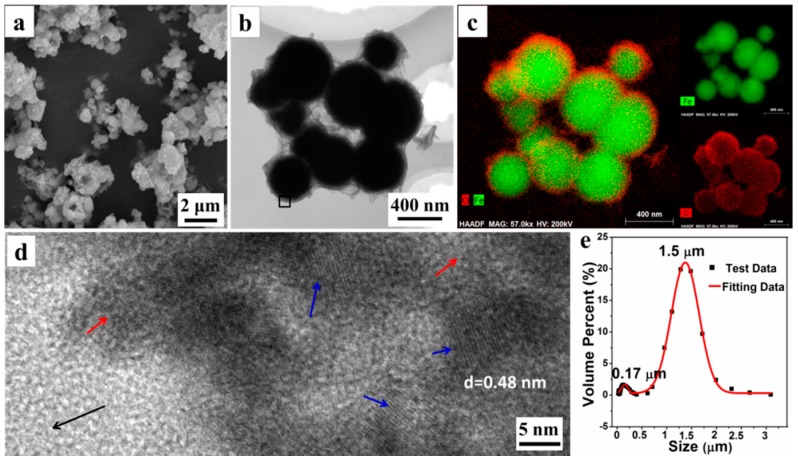
SEM (**a**); TEM (**b**); mapping images (**c**); and HRTEM (**d**) photographs (black arrow: background; red arrow: amorphous; blue arrow: crystal grains); and particle size distribution by LPSA (**e**) of Fe^0^/Fe_3_O_4_ microparticles P^0.05^_25_.

The morphology and microstructure of P^0.05^_25_ were explored by SEM, TEM, and LPSA. The SEM photograph (Figure 2a) reveals that the microparticles are obtained by the aggregation of the submicron spheres with a rough surface. Moreover, a TEM photograph (Figure 2b) shows that the single submicron sphere is wrapped in the floccules that could be organic AMPS, according to the later analysis. With the element distribution from EDXS element mapping images, shown in Figure 2c, we can see that the submicron spheres have a distinct core-shell structure, in which the interior of the sphere is rich in the Fe element, while the surface has more O elements. We believe that the core material of the core-shell structure is mainly Fe^0^, while the shell material is composed of Fe_3_O_4_ and organic matter. Simultaneously, a HRTEM image (Figure 2d) of the materials surface, taken from the black square area in Figure 2b, further shows that the surface of the submicron sphere is amorphous (the area indicated by the red arrow), whilst interspersed with a large number of Fe_3_O_4_ nanocrystals (the area indicated by the blue arrow). The interplanar crystal spacing of the nanocrystals is determined to be 0.48 nm, belonging to (111) Planes of Fe_3_O_4_. Furthermore, the LPSA analysis results (Figure 2e) also shows that there are two particle size distributions concentrated at 0.17 ± 0.05 μm and 1.50 ± 0.50 μm, respectively. Combining with the results of the SEM and TEM observations, it is confirmed that the small amount of submicron particles (about 0.17 μm) are the unaggregated Fe^0^/Fe_3_O_4_ microspheres, while the dominant micron particles (about 1.5 μm) are the aggregates of microspheres. All these results demonstrate that some submicron Fe^0^/Fe_3_O_4_ microspheres could assemble into the micron aggregates (i.e., Fe^0^/Fe_3_O_4_ microparticles) under the assistance of organic matters.

### 3.2. Effects of Organic Matters on Fe^0^/Fe_3_O_4_ Microparticles

**Figure 3:**
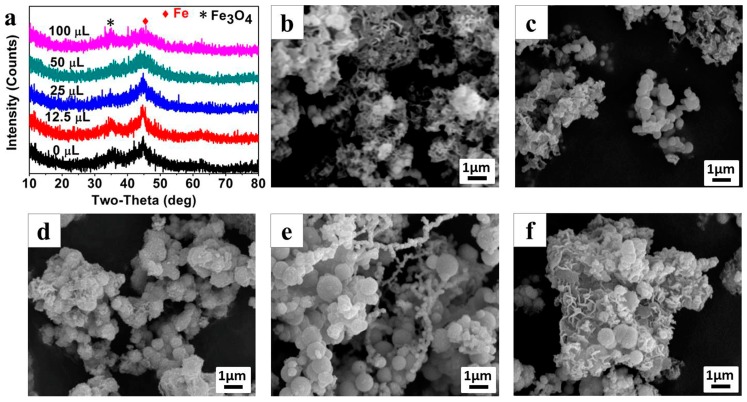
XRD patterns (**a**), SEM images (**b**–**f**) of Fe^0^/Fe_3_O_4_ microparticles synthesized under different NVP addition with a fixed AMPS addition of 0.05 g: (**b**) P^0.05^_0_, (**c**) P^0.05^_12.5_, (**d**) P^0.05^_25_, (**e**) P^0.05^_50_, and (**f**) P^0.05^_100_, respectively.

A series of experiments based on the control variable method were conducted to investigate the effects of the NVP and AMPS organic matters on the formation of the Fe^0^/Fe_3_O_4_ microparticle. The crystal compositions of the Fe^0^/Fe_3_O_4_ microparticles (P^0.05^_0_, P^0.05^_12.5_, P^0.05^_25_, P^0.05^_50_, and P^0.05^_100_) which were synthesized under different NVP additions, but with the addition of AMPS fixed at 50 mg, were determined by XRD. According to the XRD patterns (Figure 3a), similar to P^0.05^_25_, the crystal phase of other samples can all be indexed to Fe^0^ and Fe_3_O_4_. With the increase of NVP addition, the intensity of the diffraction peaks gradually decrease and the Fe^0^ phase diffraction peaks, especially at 44.7°, became wider, indicating that the growth of crystalline grains could be inhibited by NVP so that the crystallinity of the Fe^0^/Fe_3_O_4_ microparticle decreases with the increase of NVP addition.

The morphologies of Fe^0^/Fe_3_O_4_ microparticles, synthesized under different NVP additions, were observed by SEM as shown in Figure 3b–f. The Fe^0^/Fe_3_O_4_ microparticles prepared without NVP addition mainly exists in the form of floccules, and can only be observed with a few particles (Figure 3b). After introducing 12.5 μL NVP (Figure 3c), a large amount of submicron spheres, with an average diameter of about 300 nm appear, and these submicron spheres assemble into larger micron aggregates wrapped by some floccules of the polymerized AMPS. With the increase of NVP addition, the size of microspheres gradually increases, and in particular, it enlarges even to 1 μm when the NVP addition increases to 50 μL (Figure 3d,e). Thereafter, the size of the microspheres has no change even if the NVP addition is as high as 100 μL, however, a large amount of irregular sheets appeared in the aggregates, which can be inferred to be the excess NVP (Figure 3f).

**Figure 4:**
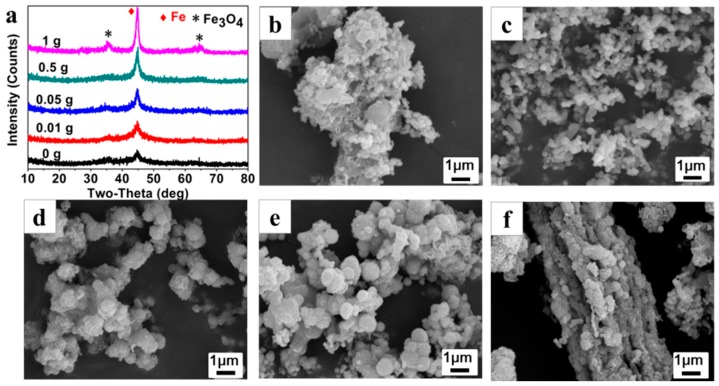
XRD patterns (**a**); SEM photographs (**b**–**f**) of Fe^0^/Fe_3_O_4_ microparticles synthesized under different AMPS additions with a fixed NVP addition of 25 μL: (**b**) P^0^_25_; (**c**) P^0.01^_25_; (**d**) P^0.05^_25_; (**e**) P^0.5^_25_; and (**f**) P^1^_25_, respectively.

Figure 4a shows the XRD patterns of the Fe^0^/Fe_3_O_4_ microparticles (P^0^_25_, P^0.01^_25_, P^0.05^_25_, P^0.5^_25_, P^1^_25_) synthesized under different AMPS additions while the NVP addition is fixed at 25 μL. As can be clearly seen, the crystal phase of the Fe^0^/Fe_3_O_4_ microparticles can be indexed to Fe^0^ and Fe_3_O_4_, but the crystallinity of Fe^0^/Fe_3_O_4_ microparticles is gradually enhanced along with the increase of AMPS additions, implying that the existence of AMPS is conducive to the assembly of the Fe^0^/Fe_3_O_4_ microspheres.

Figure 4b–f presents the SEM images of Fe^0^/Fe_3_O_4_ microparticles (P^0^_25_, P^0.01^_25_, P^0.05^_25_, P^0.5^_25_, P^1^_25_). In the absence of AMPS, the prepared precipitates are stacked loosely by blocky components of about 100 nm in size (Figure 4b). Then, some irregular aggregates composed of microspheres with a size of about 100 nm appear after adding 0.01 g of AMPS, (Figure 4c). With the increase of AMPS additions, the microsphere gradually increases to 500 nm in size and is wrapped by some floccules, meanwhile, the aggregate gradually becomes denser and larger (Figure 4d,e). Under the AMPS addition of 1 g, the microspheres are aggregated more seriously and even the rod-like aggregates that are more than 10 μm in size can be observed (Figure 4f).

The above experimental results indicate that the two organic monomers played different roles in regulating the formation of the Fe^0^/Fe_3_O_4_ microparticles, that is, the NVP is conducive to the formation of the Fe^0^/Fe_3_O_4_ microsphere, while the AMPS facilitates the assembly of the Fe^0^/Fe_3_O_4_ microsphere into aggregates (Fe^0^/Fe_3_O_4_ microparticles).

### 3.3. Regulatory Mechanism of NVP and AMPS on Fe^0^/Fe_3_O_4_ Microparticles

As mentioned above, the organic monomers NVP and AMPS play crucial roles in the formation of the Fe^0^/Fe_3_O_4_ microspheres and the assembly of the Fe^0^/Fe_3_O_4_ microspheres into Fe^0^/Fe_3_O_4_ microparticles.

NVP is a water-soluble vinyl monomer and is prone to polymerization to polyvinylpyrrolidone (PVP). The highly charged O on the carbonyl of NVP and its polymer PVP molecules can chemically bind to the metal ions in the solution [30]. Also, as a water-soluble vinyl monomer, AMPS is easily polymerized in aqueous media, and the polymeric product exhibits high temperature endurance and hydrolysis resistance. While there also exists a carbonyl group in chemical structures, the AMPS molecule is hard to bind to metal ions due to the strong salt resistance resulting from the salt-insensitive sulfonic acid group [31].

**Figure 5:**
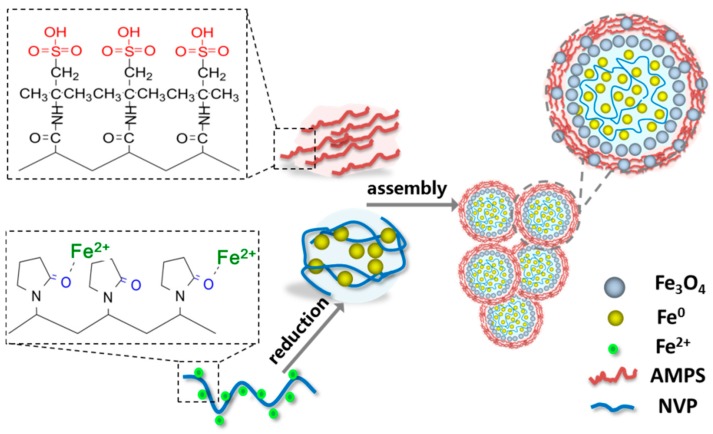
Diagrammatic sketch for speculative regulatory mechanism of NVP and AMPS on the formation of Fe^0^/Fe_3_O_4_ microparticles.

When the iron chloride tetrahydrate, NVP, and AMPS were dissolved together in aqueous solution, the ferrous ions were preferentially coordinated with the carbonyl (C=O) on NVP, meanwhile the NVP molecules were gradually polymerized. Subsequently, the coordinated ferrous ions were reduced by sodium borohydride, leading to the heterogeneous nucleation and the growth of the Fe^0^ grain. Because of the binding effect of NVP molecules, the Fe^0^ grain growth was greatly restrained. Upon the polymerization of NVP, a lot of nano-Fe^0^ grains, bound to NVP, could be aggregated together into submicron Fe^0^ microspheres mingled with a NVP polymer. On the other hand, the gradually polymerized AMPS were wrapped outside the Fe^0^ microspheres, and a number of the submicron Fe^0^ microspheres could be assembled into the Fe^0^ microparticles in the micron scale during the further polymerization of AMPS. In addition, the slight oxidation might take place on the surface of the formed Fe^0^ microspheres in the atmosphere so as to obtain Fe^0^/Fe_3_O_4_ microparticles. This regulatory mechanism is consistent with the experimental results about the effects of NVP and AMPS on Fe^0^/Fe_3_O_4_ microparticles. Therefore, the proposed regulatory mechanism of NVP and AMPS on the formation of Fe^0^/Fe_3_O_4_ microparticles is illustrated in Figure 5.

**Figure 6:**
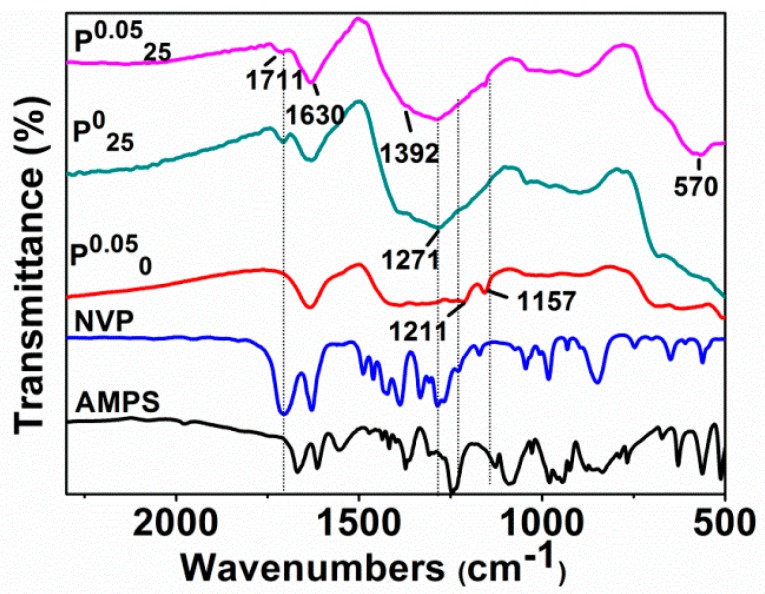
FTIR spectra of AMPS, NVP, and Fe^0^/Fe_3_O_4_ microparticles synthesized by adding different organic matters: AMPS, NVP, P^0.05^_0_, P^0^_25_, and P^0.05^_25_ from the bottom up.

Figure 6 shows the infrared absorption spectra of AMPS, NVP, and Fe^0^/Fe_3_O_4_ microparticles including P^0.05^_0_, P^0^_25_, and P^0.05^_25_ synthesized under different additions of NVP and AMPS. Clearly, the C–N absorption band of NVP at 1285 cm^−1^, which is absent in the spectrum of P^0.05^_0_, but appears in P^0^_25_ and P^0.05^_25_. Similarly, the characteristic bands of AMPS, such as S=O symmetric stretching vibration band and C–N stretching vibration band at 1211 cm^−1^ and 1157 cm^−1^, are not observed in the spectrum of P^0^_25_, but appear in P^0.05^_0_ and P^0.05^_25_. These results agree with the experiments, whether the NVP and AMPS were added in or not.

It is worth noting that the absorption band at about 1711 cm^−1^, belonging to C=O, disappears in the spectrum of P^0.05^_0_ (AMPS added only), but appears with a relatively weak intensity in the spectra of P^0^_25_ (NVP added only) and P^0.05^_25_ (both NVP and AMPS added). It is well known that the reductant NaBH_4_ used in our synthesis exhibits the characteristic of selectively reducing ketones. The unprotected carbonyl groups on AMPS and NVP were likely to be reduced to hydroxyl groups in the preparation of the Fe^0^/Fe_3_O_4_ microparticles. Actually, the coordinated ferrous ions can protect these carbonyl groups from reduction. The carbonyl groups in AMPS were completely reduced by NaBH_4_, while the carbonyl groups in NVP bound to Fe^2+^ had avoided the reduction. However, the absorption band belonging to C=O is also weakened due to its coordination with ferrous ions. These results also prove that NVP is able to directly regulate the formation of the Fe^0^/Fe_3_O_4_ microspheres by binding to ferrous ions, and AMPS is involved in the regulatory aggregation of the Fe^0^/Fe_3_O_4_ microparticles.

### 3.4. Morphology and Magnetism of Macroporous Fe_3_O_4_ Microparticles

**Figure 7:**
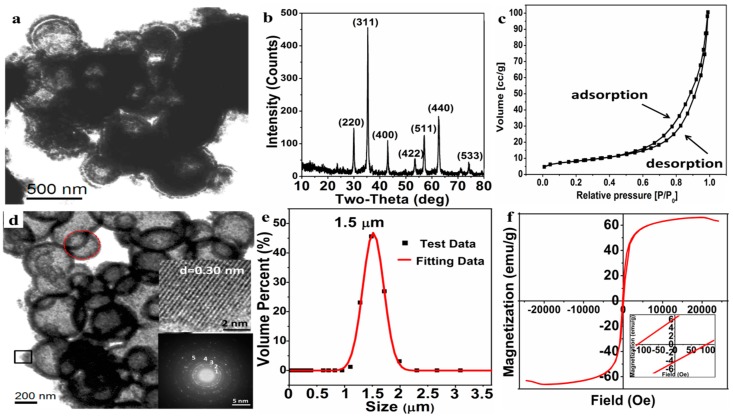
TEM image of I^0.05^_25_ before calcination (**a**); XRD pattern (**b**); nitrogen adsorption-desorption curves (**c**); TEM image (**d**); particle size distribution (**e**); and magnetic hysteresis loop (**f**) of macroporous Fe_3_O_4_ microparticles F^0.05^_25_ after calcination.

After undergoing the solvothermal reaction, the Fe^0^/Fe_3_O_4_ microparticles were converted to the Fe_3_O_4_ microparticles wrapped by organic matters (Figure 7a). It can be seen that the hollow structure of microsphere is basically formed and the microspheres are still wrapped by a floccule layer of organic matters.

The macroporous Fe_3_O_4_ microparticles were obtained after calcination to remove the organic matters under a protective atmosphere. The phase composition and morphology of the macroporous Fe_3_O_4_ microparticles were characterized by XRD, TEM, and LPSA. According to the XRD pattern (Figure 7b), the well-resolved diffraction peaks are well indexed to Fe_3_O_4_ (JCPDS NO. 88-0866), and the crystal exhibits a high crystallinity. Nitrogen adsorption-desorption curves (Figure 7c), a type IV isotherm, show that the Fe_3_O_4_ has a stacked pore structure and the multi-point BET specific surface area is estimated to 29.23 m^2^/g, and the BJH cumulative total pore volume (d > 2 nm) is 0.16 cc/g. As for the specific porous structure, it can be further and clearly seen from the TEM image (Figure 7d), in which the macroporous Fe_3_O_4_ microparticle is assembled from a number of open-cell hollow microspheres. The pore size of the Fe_3_O_4_ microparticle is mainly distributed in 300–500 nm, while the porosity of the Fe_3_O_4_ microparticle can be reasonably attributed to both the open-cell hollow of microspheres and the gaps between the microspheres, as shown in Figure 7d. The HRTEM image of Fe_3_O_4_ nanoparticles (the right middle insert in Figure 7d), from the shell of open-cell hollow sphere by the black square area, shows that the interplanar crystal spacing is about 0.30 nm, corresponding to the (220) plane of Fe_3_O_4_. In addition, the main polycrystalline diffraction rings (marked as 1, 2, 3, 4, 5), which correspond to the (220), (311), (400), (511), and (440) crystal planes of the Fe_3_O_4_, respectively, can be clearly observed in the selected area electron diffraction pattern (the right bottom insert in Figure 7d) from the red round area—this is consistent with the XRD results. The particle size distribution by LPSA (Figure 7e) reveals that the average size of the Fe_3_O_4_ microparticles is about 1.50 ± 0.50 μm with a PDI 0.218, indicating that the prepared macroporous Fe_3_O_4_ microparticles have a good dispersibility in the aqueous phase and less agglomeration in application.

The magnetic hysteresis loop by VSM (Figure 7f) verifies that the prepared macroporous Fe_3_O_4_ microparticles exhibits ferromagnetism with a high saturation magnetization (Ms = 66.14 emu/g), remnant magnetization (Mr = 6.33 emu/g), and coercivity (Hc = 105.32 Oe). Such a ferromagnetic macroporous material is expected to be applied as a drug-loaded material and to provide a heat source for the temperature-sensitive material by applying an alternating magnetic field.

Similar to F^0.05^_25_, after subsequent NKE controlled oxidation, Fe^0^/Fe_3_O_4_ microparticles with different diameters of spheres will be converted into macroporous Fe_3_O_4_ particles with different pore sizes and particle sizes. The macroporous Fe_3_O_4_ particles with such a large pore structure and ferromagnetic properties may provide a good carrier and heat source for the magnetocaloric-responsively controlled drug release system.

### 3.5. A Preliminary Discussion on Holing Mechanism of Open-Cell Hollow Microsphere

More interestingly, many open-cell hollow microspheres appeared after undergoing the solvothermal reaction and calcination (F^0.05^_25_). This phenomenon would be of great significance to the controllable synthesis of various hollow microspheres. On the basis of a comprehensive analysis on the synthesis process, probable holing mechanisms of open-cell hollow microspheres would be preliminarily suggested.

**Figure 8:**
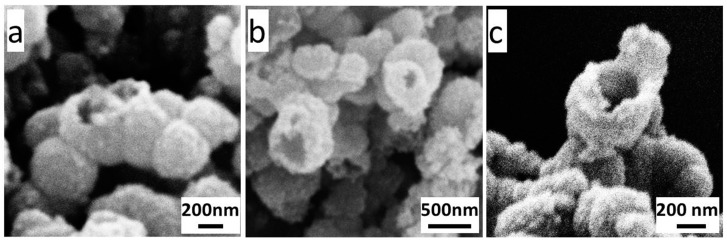
SEM local magnification images of macroporous Fe_3_O_4_ microparticles F^0.05^_25_: dumbbell-like or bead-like hollow microspheres (**a**); expanding holes (**b**); and typical open-cell hollow microsphere (**c**).

One of the probable holing mechanisms could be related to the thermal stress effect. In the Kirkendall controlled oxidation process, the contacted microspheres would be locally fused together due to the sustained grain growth in the adjacent region to form the dumbbell-like or bead-like hollow microspheres (shown in Figure 8a). The neck of the dumbbell-like or bead-like hollow microsphere was prone to fracture due to the thermal stress on the interface under an elevated temperature, and a hole was left on the shell. This holing process might occur during either the solvothermal reaction or the calcination process.

Another probable holing mechanism could be the surface tension mechanism. As mentioned earlier, a large amount of NVP was mingled in the obtained Fe^0^/Fe_3_O_4_ microspheres, and remained in the cavity during the cavitation process. At a high temperature, the vaporized NVP could escape out of the microsphere through the looser part of the shell, leaving behind an initial hole there. Due to the high surface energy resulting from the small radius of curvature at the edge of the initial hole, the surficial atoms could continuously diffuse inward, the shell driven by the surface tension, and thus the hole could be gradually enlarged. This holing way mainly occurred in the calcination process. Some probable holes being enlarged are shown in Figure 8b, and a typical open-cell hollow microsphere is shown in Figure 8c. All these images in Figure 8 are taken from sample F^0.05^_25_.

Certainly, the above suggestions should be supported by sufficient experimental results, and further theoretical calculations and experimental verifications are still ongoing in our laboratory.

## 4. Conclusions

In this study, a kind of macroporous magnetic Fe_3_O_4_ microparticles composed of the aggregates of Fe_3_O_4_ open-cell hollow microspheres was successfully prepared by a novel organic matter assisted open-cell hollow sphere assembly method. With the help of the specific affinity of ferrous ions to carbonyl groups on NVP and the polymerization of organic monomers, the reduced irons underwent heterogeneous nucleation and grain growth to form the Fe^0^/Fe_3_O_4_ microspheres consisting of a lot of nano-Fe^0^ grains. Then, they were assembled into Fe^0^/Fe_3_O_4_ microparticles wrapped by organic matters. The NVP can promote the formation of the Fe^0^/Fe_3_O_4_ microsphere via bonding to ferrous ions and self-polymerizing, while the AMPS can facilitate the assembly of the Fe^0^/Fe_3_O_4_ microsphere into larger aggregates (Fe^0^/Fe_3_O_4_ microparticles) via enwrapping around the microsphere during the polymerization. After the Kirkendall controlled oxidation had finished, the Fe^0^/Fe_3_O_4_ microparticles were converted to the macroporous Fe_3_O_4_ microparticles (the aggregates of a number of the hollow/open-cell hollow Fe_3_O_4_ microsphere). The unique porous structure of the macroporous Fe_3_O_4_ microparticle is mainly derived from both the open-cell hollow of microspheres and the gaps between the microspheres. While the research on open-cell hollow microspheres was very preliminary and the holing mechanism should also be further studied, the strategy to assemble the open-cell hollow Fe_3_O_4_ microspheres into the macroporous microparticles has been verified as feasible. Additionally, the prepared macroporous magnetic Fe_3_O_4_ microparticles were expected to have broad application prospects on magnetocaloric-responsively controlled drug release systems.
